# A High-Throughput Assay for Quantifying Phenotypic Traits of Microalgae

**DOI:** 10.3389/fmicb.2021.706235

**Published:** 2021-10-06

**Authors:** Phoebe A. Argyle, Jana Hinners, Nathan G. Walworth, Sinead Collins, Naomi M. Levine, Martina A. Doblin

**Affiliations:** ^1^Climate Change Cluster, University of Technology Sydney, Ultimo, NSW, Australia; ^2^Institute of Coastal Ocean Dynamics, Helmholtz-Zentrum Geesthacht, Geesthacht, Germany; ^3^Department of Biological Sciences, University of Southern California, Los Angeles, CA, United States; ^4^Institute of Evolutionary Biology, University of Edinburgh, Edinburgh, United Kingdom; ^5^Sydney Institute of Marine Science, Mosman, NSW, Australia

**Keywords:** microalgae, high-throughput approaches, phenomics, trait-based approaches, diatoms, climate modelling, biogeochemical cycles

## Abstract

High-throughput methods for phenotyping microalgae are in demand across a variety of research and commercial purposes. Many microalgae can be readily cultivated in multi-well plates for experimental studies which can reduce overall costs, while measuring traits from low volume samples can reduce handling. Here we develop a high-throughput quantitative phenotypic assay (QPA) that can be used to phenotype microalgae grown in multi-well plates. The QPA integrates 10 low-volume, relatively high-throughput trait measurements (growth rate, cell size, granularity, chlorophyll a, neutral lipid content, silicification, reactive oxygen species accumulation, and photophysiology parameters: ETRmax, I_k_, and alpha) into one workflow. We demonstrate the utility of the QPA on *Thalassiosira* spp., a cosmopolitan marine diatom, phenotyping six strains in a standard nutrient rich environment (f/2 media) using the full 10-trait assay. The multivariate phenotypes of strains can be simplified into two dimensions using principal component analysis, generating a trait-scape. We determine that traits show a consistent pattern when grown in small volume compared to more typical large volumes. The QPA can thus be used for quantifying traits across different growth environments without requiring exhaustive large-scale culturing experiments, which facilitates experiments on trait plasticity. We confirm that this assay can be used to phenotype newly isolated diatom strains within 4 weeks of isolation. The QPA described here is highly amenable to customisation for other traits or unicellular taxa and provides a framework for designing high-throughput experiments. This method will have applications in experimental evolution, modelling, and for commercial applications where screening of phytoplankton traits is of high importance.

## Introduction

Microalgae are ubiquitous in marine and freshwater ecosystems and play a critical role by driving the cycling of many elements, thereby regulating water quality (Falkowski et al., 2008). In addition to being genetically diverse, phytoplankton taxa show high inter- and intra-specific phenotypic diversity (Malcom et al., 2015; Hattich et al., 2017; Brandenburg et al., 2018). Understanding how phytoplankton traits vary within a given taxon or genotype across environments (phenotypic plasticity) and constraints on this variability (correlations or trade-offs between trait values) is essential for understanding phytoplankton functions, marine ecosystem dynamics, and rates of biogeochemical cycling.

In addition to their roles in aquatic ecosystems, microalgae are used in a wide range of commercial settings including nutraceutical development (Chacón-Lee and González- Mariño, 2010), drug discovery (Lauritano et al., 2016; Martínez Andrade et al., 2018), biofuels (Chisti, 2007; Talebi et al., 2013; Rajak et al., 2019), and wastewater treatment (Abdelaziz et al., 2014; Piligaev et al., 2018). In these industries, phenotyping methods (phenomics) are often used to screen species or strains for specific purposes such as for use in biofuels (Nascimento et al., 2013; Talebi et al., 2013) or production of bioactive compounds that could be used in human medicine (Lauritano et al., 2016). Such methods are less commonly used in exploratory ecological research.

In both applied and research settings, phytoplankton phenotypes are typically defined by traits such as population growth rate, cell size, or nutrient acquisition (Litchman and Klausmeier, 2008; Edwards et al., 2011). Population growth rate is arguably the most universally measured trait, with high-throughput proxies widely applied including changes in *in vivo* fluorescence or optical density, both of which can be quantified in a well-plate format (Gross et al., 2018). Cell size is considered a “master trait” in phytoplankton ecology due to its influence on nutrient or light aquisition, sinking rates, and predation risk (Litchman and Klausmeier, 2008; Finkel et al., 2010). In addition to population growth and cell size, measuring components of cells such as pigments, lipids, proteins, or toxins can give indications about the physiological state of the cell, as well as being target compounds for commercial extraction.

Traits are not independent, and one emerging goal of phenomics is to capture multi-dimensional phenotypes, not just individual traits (Houle et al., 2010). Trait combinations manifest into different growth strategies, allowing population increase or persistence in the environment. However, due to trade-offs between traits, there are potential limitations on the feasible trait combinations that can manifest both as plastic and evolutionary responses. These limitations on phenotypic expression may shape trajectories along which phytoplankton may evolve (Hinners et al., in prep) and can be used to inform more nuanced, trait-based models of phytoplankton evolution (Walworth et al., 2021).

Quantifying phenotypic plasticity in microalgae has typically been very labour intensive, requiring high numbers of replicate cultures of different strains to be grown in different environments. In addition, many current methods for quantifying trait values are often time intensive (e.g., microscopy for cell counts or sizing) or require high biomass (e.g., cellular stoichiometry), which limits the number of experimental units that can be measured in any one study. Thus, so far, phenotyping has focused on either a few traits in multiple environments or strains (Malcom et al., 2015; Brennan et al., 2017; Barton et al., 2020) or multiple traits in few environments or strains (Lindberg and Collins, 2020; Ajani et al., 2021) due to constraints regarding space, time, or funds. These constraints limit extensive multiple driver experiments in the context of global ocean change (Boyd et al., 2018). Here we develop a quantitative phenotyping assay that allows multiple traits relevant to phytoplankton physiology, ecology, and evolutionary studies to be measured in multiple strains and environments with relatively high throughput. By miniaturising phenotyping methods, we create a workflow that reduces costs and resource requirements for trait-based experiments.

The Quantitative Phenotypic Assay (QPA) developed in this study allows for the higher-throughput characterisation of integrated phenotypes (multiple traits quantified simultaneously). We extended the work of Gross et al. (2018) who developed a high-throughput methodology for estimating growth of chain-forming diatoms in well-plates and demonstrate that nine additional traits can be quantified simultaneously by one researcher with three pieces of standard laboratory equipment: a flow cytometer, plate reader, and fluorometer. The traits measured are a combination of effect and response traits (Litchman et al., 2015) including resource acquisition traits (light utilisation, silicification) and other traits that can be used to infer the physiological status of cells. The QPA collects all trait data from the same experimental unit/culture, providing greater statistical robustness than studies that compile trait data from multiple cultures. Depending on the growth environment used, trait data can be collected within 7–10 days of inoculation by one researcher and quantification does not require bulk culturing or harvesting. The QPA allows comparison of multiple traits across large numbers of strains or growth environments, and experimentally captures multi-directional trade-offs between traits. This method has potential for wide applicability to ecology, evolutionary biology, and biotechnology research and provides a framework for the inclusion of new traits and taxa.

## Materials and Equipment

### Microalgae Strains and Culturing Conditions

The QPA was developed using six strains of *Thalassiosira* from the Provasoli-Guillard National Center of Marine Phytoplankton (NCMA, formerly known as the CCMP, https://ncma.bigelow.org/; Table 1), in addition to five new isolates of centric diatoms established from an ocean observatory in Australia's Integrated Marine Observing System. Diatoms are important for biogeochemical cycling of multiple elements (silica in particular) and *Thalassiosira* is a highly diverse and cosmopolitan genus (Malviya et al., 2016). Stock cultures of microalgae were maintained in 50 mL polystyrene tissue culture flasks (Falcon^®^ Corning, NY, USA) in artificial seawater (hereafter called ASW; hydrous and anhydrous salts only from ASP-M medium, ncma.bigelow.org) with f/2 nutrients (Guillard, 1975) at 20°C, with 60 μmol m^−2^s^−1^ of light on a 12:12 light cycle. Flasks were incubated standing upright due to space limitations. Although most strains were uniform cultures of single cells, some strains exhibited clumping and chain formation at different times during their maintenance.

**Table 1:** Domesticated *Thalassiosira* strains used to develop and test the QPA, including species identification and location collection.

**Species**	**Strain code**	**Collection location**
*Thalassiosira pseudonana*	CCMP3367	44.9335° N 12.7005° E North Adriatic Sea
*Thalassiosira weissflogii*	CCMP1010	37° N 65° W, Gulf Stream, between Bermuda and New York (very approx)
*Thalassiosira weissflogii*	CCMP1050	32.966° N 117.251° W, Del Mar Slough, California USA
*Thalassiosira rotula*	CCMP3264	40.49° N 14.15° E, Marechiara, SZN long term sampling station, Gulf of Naples, Italy
*Thalassiosira* sp.	CCMP1059	19.665° N 156.034° W Aquaculture Pond, Oahu, Hawaii USA (approx.)
*Thalassiosira* sp.	CCMP2929	Unknown

### Laboratory Equipment

Cultures were incubated in temperature-controlled incubators (Aralab, Albarraque, Portugal). A plate reader (TECAN Infinite M1000 Pro, Männedorf, Switzerland) was used for *in vivo* fluorescence measurements and for the reactive oxygen species assay. Flow cytometry was conducted using a Cytoflex LX (Beckman Coulter, CA, US). Photophysiology measurements were done using a WATER-PAM (Walz GmbH, Effeltrich, Germany).

### Culturing Materials and Consumables

- Multi-well polystyrene plates: 12, 24, and 48-wells (Corning Costar, Acton, MA).
- Breathable Plate seals (Breathe-Easy, Diversified Biotech).

### Chemicals and Solutions

- Artificial seawater.
- F/2 nutrient stocks (to modify nutrient levels).
- Nutrient stocks (to modify nutrient levels).
- 8% Paraformaldehyde solution to fix samples (Electron Microscopy Sciences, Ft Washington, PA).
- For the silicification assay, PDMPO (LysoSensor™ Yellow/Blue DND-160, Invitrogen) purchased at a concentration of 1 mM diluted in Milli-Q water to create a stock solution of 12.5 μM.
- For neutral lipid measurements, BODIPY™ 505/515 (4,4-difluoro-1,3,5,7-tetra-methyl-4-bora-3a,4a-diaza-s-indacene; Thermo Fisher, MA, USA). This was purchased as a powder and mixed with DMSO to create a stock solution of 2 mg mL^−1^.
- For measurement of ROS, 2′,7′-dichlorodihydrofluorescein diacetate (H_2_DCFDA; Thermo Fisher, MA, SA) (Knauert and Knauer, 2008; Szivák et al., 2009). Purchased as a powder, a stock solution of 2.5 mg mL^−1^ H_2_DCFDA was made in DMSO and stored at −20°C in the dark.

## Methods

### QPA Trait Selection

This assay was developed to allow for the high-throughput phenotyping of microalgae using a large enough number of simultaneously quantified traits to capture multi-variate phenotypes with enough resolution to distinguish different strains. For ease of integration into a single workflow, all traits needed to be quantifiable from low-volume cultures grown in multi-well plates. Key considerations for trait choice were their biogeochemical or ecological relevance, speed of quantification, and the volume or biomass required for measurement. We also wanted to include traits that were statistically orthogonal to one another (i.e., were not consistently correlated/covarying) to capture different aspects of phenotypic variability. Orthogonality was tested during pilot studies by determining correlations between trait values using principal component analysis (PCA). The suite of traits presented here can be expanded upon in the future as new technological advances are made which enable the quantification of additional traits utilising small volumes.

Traits that allowed for high-throughput (minimal handling and analysis time), miniaturisation (low density or biomass requirements), use of standard rather than specialist laboratory equipment (i.e., plate reader, flow cytometer), and were informative about basic cellular and biological function, particularly resource acquisition were included (Supplementary Table 1). Growth rate and cell size were the most biogeochemically relevant, with photophysiological parameters (alpha, I_k_, and ETRmax) and silicification being resource acquisition traits that could be directly incorporated into biogeochemical models (Dutkiewicz et al., 2013). Response traits comprised relative chlorophyll-a content, relative lipid content and the more general trait of cell granularity (SSC), as well as production of reactive oxygen species (ROS), an indicator of cell stress (McInnes et al., 2019). These traits capture key biological functions of growth, photosynthesis, silica uptake and oxidative stress, which occur in tandem within diatoms to maintain cellular function and may be modified in response to the environment.

### QPA Workflow

The QPA is initiated by inoculating multi-well plates (12 wells, 4.4 mL per well, Corning CoStar, MA, USA) with a starting concentration of ~2,000 cells mL^−1^. Multi-well culture plates are sealed with gas-permeable membranes (Breathe-Easy, Diversified Biotech, MA, USA) to prevent evaporation and cross-contamination before replacing the lids. These seals may also be used in place of plastic lids. Following the incubation period, traits are measured during exponential, asexual population growth, so that trait values reflect those of actively growing and dividing cells. To determine the timing for trait quantification, *in vivo* Chl-a fluorescence of the cultures is monitored daily at a consistent point in the photoperiod (see below) to observe growth and determine when mid-exponential phase is reached. For some environments, especially when growth or carrying capacity is limited, the exponential phase can be short (1 or 2 days), leading to trait measurements being made at early stationary phase.

**Figure 1:**
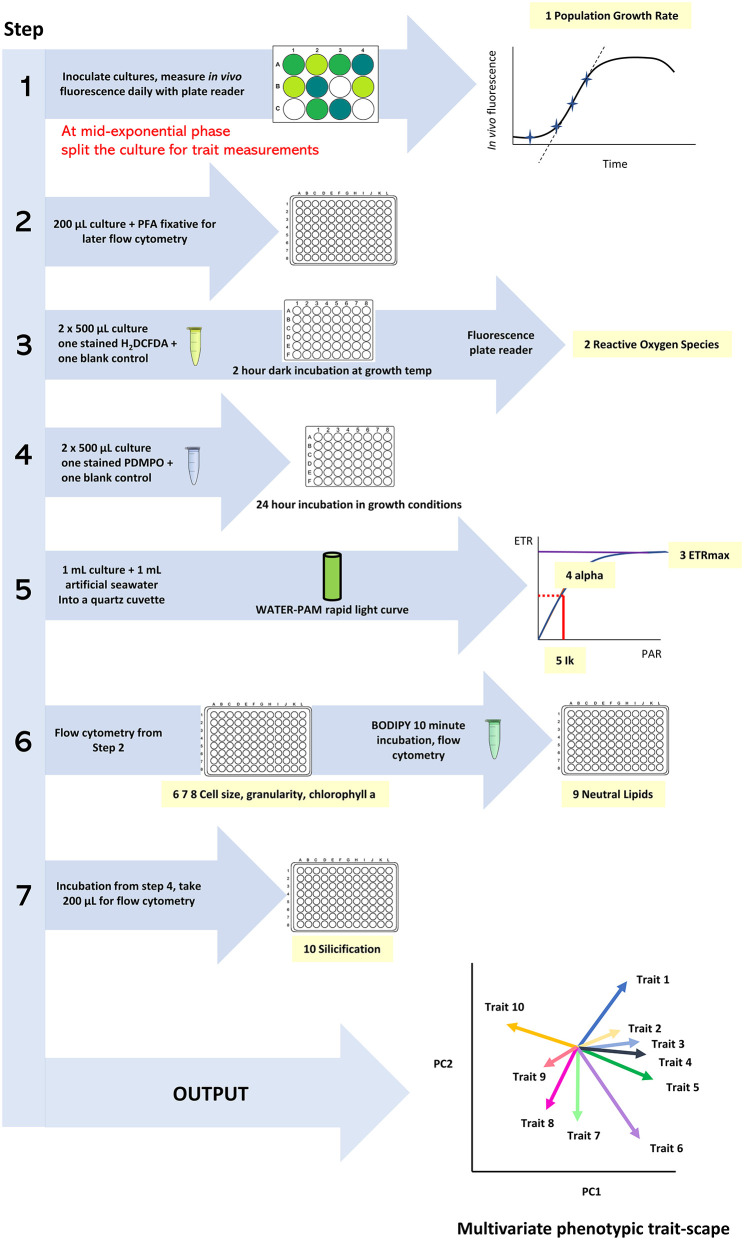
The workflow of the Quantitative Phenotyping Assay (QPA) outlining the sequence of actions, measurements, and data outcomes.

All traits are assessed on the same day, with the total volume from each culture well split across the different trait measurements (Figure 1). The QPA workflow involves coordinated sampling with timed stain incubations and time-sensitive photophysiology measurements. Trait measurements are conducted in the same order to create a streamlined workflow and involve the following steps:

1. fixing aliquots for flow cytometry traits (200 μL, cell size, granularity, Chl-a, neutral lipids),
2. initialising the ROS assay (1 mL, 2 h incubation),
3. initialising the silicification assay (1 mL, 24 h incubation),
4. measuring the photophysiology traits measured (1 mL, ~1 min per measurement) and
5. measuring flow cytometry traits (1–3 h duration depending on sample number).

Flow cytometry analyses are most efficient if run in the afternoon of the day of sampling, however these samples can also be snap frozen in liquid nitrogen, stored at −80°C and analysed in delayed mode.

To conduct the full 10-trait assay in multiple environments, it would first be necessary to calculate the maximum number of culture wells that can be realistically harvested per day by the researcher(s). For example, if one WATER-PAM instrument is available, we recommend no more than 60 wells be harvested on a single day, to obtain photophysiological measurements within a 2-h window to control for diel effects. This may require some estimation on the part of the researcher, as it is not always possible to predict which wells will be ready for harvest on any 1 day. We suggest pilot trials that only measure growth would be prudent (see discussion) or inoculating an experiment in a staggered way to manage the workload across harvest days.

### Trait Measurement Methods

#### Growth Rate

*In vivo* fluorescence of Chl-a is a proxy for cell density and used to estimate population growth rates (Wood et al., 2005). Each day, 3 h after the onset of the photoperiod, Chl-a fluorescence of the growth cultures is measured using a plate reader (TECAN Infinite M1000 Pro, Männedorf, Switzerland) with a 455/680 nm excitation/emission filter. Maximum growth rates are calculated using a linear regression of natural log fluorescence values over four time points. If exponential growth is shorter, then a minimum of two time points are used.

#### Flow Cytometry Traits

The following traits are measured simultaneously on a flow cytometer (Cytoflex LX, Beckman Coulter, CA, US) from a 200 μL aliquot of culture fixed with 20 μL paraformaldehyde (0.8% v/v final concentration, Electron Microscopy Sciences, Ft Washington, PA): cell size and granularity, pigment content, and lipid content. Culture aliquots are transferred from the culture vessel into a 96-well round-bottom plate (Corning Costar, MA, USA) before fixation. Fluorescent calibration beads (CytoFlex Daily QC beads, Beckman Coulter, CA, USA) are used to calibrate the instrument prior to use and bead fluorescence is used to standardise other traits to obtain comparable results between experiments.

#### Cell Size and Granularity

Cell size is estimated using forward scatter (FSC) values. The diatom population is differentiated from the background and cell debris using minimum Chl-a and forward scatter signal thresholds. Spherical plastic beads of known diameters (2, 4, 6, 10, 15 μM; Invitrogen, CA) are used to correlate median FSC (measured for at least 200 cells, but usually >1,000 cells in dense populations) to size “equivalent spherical diameter.” For the flow cytometer used in developing the QPA, the equation was Size = (FSC + 275,549)/83,539. As *Thalassiosira* are centric diatoms, they are “pill box” shaped, and thus not truly spherical, however here we use FSC to approximate diameter and assess relative changes in cell size. Side scatter, which is an indication of cell complexity or granularity, is also measured and may give an indication about the physiological state of the cell (Pereira et al., 2011).

#### Pigment Content

Relative Chl-a content is measured as the median fluorescence of cells using 488 nm excitation, 690/50 nm detection. Cell size effects on Chl-a content were accounted for by dividing the median cellular Chl-a value of the population by the estimated median cell size. In pilot studies Chl-a fluorescence was observed to degrade with storage in pilot studies, as has been documented elsewhere (Marie et al., 2005). It is recommended to analyse samples on the same day as collection and not mix fresh and fixed stored samples within one study.

#### Neutral Lipids

Neutral lipids are quantified using BODIPY™ 505/515 (4,4-difluoro-1,3,5,7-tetra-methyl-4-bora-3a,4a-diaza-s-indacene; Thermo Fisher, MA, USA). The fluorescence of unstained cells is measured on each sample using 488 nm excitation and 525/40 nm detection. This is done in tandem with the flow cytometry measurements for cell size, granularity and Chl-a. After background fluorescence has been quantified, a stock solution of 2 mg mL^−1^ BODIPY 505/515 in DMSO is added to fixed samples (0.8% paraformaldehyde v/v final concentration) in a 1/1,000 dilution, resulting in a final concentration of 2 μg mL^−1^. After staining for 10 min in the dark, fluorescence was measured again on the flow cytometer. Neutral lipid content per cell is calculated as the difference in median fluorescence between pre- and post-stained cells. BODIPY has been shown to be less susceptible to fading [compared with Nile Red, also used as a lipid stain (Brennan et al., 2012)]. We recommend to not mix fresh and frozen samples for phenotyping, as it is unknown how freezing and thawing affects the permeability of fixed cells for subsequent staining.

#### Silicification via PDMPO Fluorescence

The fluorescent dye PDMPO (2-(4-pyridyl)-5-[(4-(2-dimethylaminoethylamino carbamoyl)methoxy)phenyl]oxazole) has been used in several studies to quantify silicification in both diatom cultures and field populations (Shimizu et al., 2001; Leblanc and Hutchins, 2005; Mcnair et al., 2015; Baker et al., 2016; Petrou et al., 2019), as it is taken up into the silica deposition vesicles and then incorporated into newly deposited frustules (Shimizu et al., 2001). In field studies, mixed populations are incubated for up to 24 h followed by PDMPO quantification using fluorescence microscopy or chemical extraction (Mcnair et al., 2015). In this assay, we use flow cytometry to quantify PDMPO uptake per cell following a 24-h incubation with PDMPO. To allow for high-throughput quantification, we analysed PDMPO uptake using flow cytometry which provides an indication of overall silica uptake (Baker et al., 2016). Note that quantification with flow cytometry does not differentiate between vacuolar and frustule-incorporated silica.

To initiate the assay, duplicate 500 μL aliquots of culture are transferred to a 48-well plate (Falcon, Corning, NY), one to act as a blank control, the other to be treated with the dye. PDMPO (LysoSensor™ Yellow/Blue DND-160, Invitrogen) at a concentration of 1 mM is diluted in Milli-Q water to create a stock solution of 12.5 μM. Five μL of this stock is added to each sample well, resulting in a final concentration of 0.125 μM. Plates are sealed (Breathe-Easy, Diversified Biotech) and incubated under growth conditions for 24 h. The incubation is started within 1 h of the daily *in vivo* fluorescence measures, between 3 and 4 h after the onset of the 12-h light-period. After 24 h cells are analysed flow cytometrically to observe the difference in median fluorescence between the stained and unstained cells. Unstained cells are used to quantify background fluorescence in the near UV channel (375 nm excitation/490–530 nm emission). Due to cell division, occasionally double peaks in fluorescence are observed (Supplementary Figure 1). This phenomenon is accounted for by using the median change in PDMPO fluorescence (T_24_-T_0_ fluorescence to account for background) across the whole population and dividing by the growth rate over the 24 h incubation time. As PDMPO is a fluorescent dye, it degrades over time and is altered by the addition of fixatives. For this reason, all flow cytometry measurements should be done on live cells. Light exposure during the incubation also causes the dye to degrade. As the cells need light to grow it is not possible to conduct this assay in the dark, therefore we recommend comparing trait values of cultures grown under the same light conditions to limit this source of variability. Similar caution should be taken when using fluorescent dyes for other trait measurements.

#### Photophysiological Traits

Photophysiological traits are determined with a WATER-PAM (Walz GmbH, Effeltrich, Germany). One mL of culture is diluted with 1 mL of sterile artificial seawater in a quartz cuvette and assessed using the rapid light protocol in the WinControl software (Ralph and Gademann, 2005). The protocol exposes the cells to eight steps of increasing irradiance (between 0 and 1,878 μmol m^−2^ s^−1^) for 10 s to assess the effective quantum yield at each step. The gain was set at 5, with a saturation pulse width of 0.8. The resulting parameters from the curve are the maximum electron transport rate (ETRmax), alpha (photosynthetic rate during light-limitation), and the minimum saturating irradiance in μmol m^−2^ s^−1^ (I_k_). In the case of an over-saturated signal, a 25% rather than a 50% dilution of culture is used.

#### Reactive Oxygen Species

Production of reactive oxygen species (ROS) is measured using the fluorescent probe 2′,7′-dichlorodihydrofluorescein diacetate (H_2_DCFDA; Thermo Fisher, MA, SA) (Knauert and Knauer, 2008; Szivák et al., 2009). This stain is taken up and hydrolyzed within the cells to a non-fluorescent compound 2′,7′dichlorodihydrofluorescein (H_2_DCF). In the presence of ROS and cellular peroxidases, H_2_DCF is converted to 2,7′dichlorofluorescein (DCF) which is highly fluorescent. A stock solution of 2.5 mg mL^−1^ H_2_DCFDA is made in DMSO and stored at −20°C in the dark. Two 500 μL aliquots of experimental culture are transferred to a 48-well tissue culture plate; 2 μL of stain is added to one aliquot, with the other acting as a blank. The plates are sealed (Breathe-Easy, Diversified Biotech), wrapped in aluminium foil, and incubated in the dark at growth temperature for 2 h. Incubation is done in the dark due to the effects of light on the dye itself (Knauert and Knauer, 2008). Fluorescence of H_2_DCFDA is read using a plate reader with 488 nm excitation 525 nm emission (TECAN Infinite M1000 Pro, Männedorf, Switzerland). ROS concentration is calculated as the difference in fluorescence units between the stained and unstained aliquots of each culture. The number of cells in the 500 μL aliquot is determined from the flow cytometry trait analyses, from which an RFU per cell for ROS could be calculated. This value was then normalised to cell size by dividing the RFU per cell by the median cell size in μm.

### Experiment Details: Culturing Conditions

Following integration of the trait measurements into a single workflow, the QPA was evaluated under several culturing conditions. Our first set of experiments aimed to demonstrate the use of the QPA on *Thalassiosira* strains grown in “standard” growth conditions and test the effects of miniaturised culturing on trait values. We then tested the efficacy of the QPA to detect phenotypic plasticity using four strains grown under 36 different environments. Finally, to assess the use of the QPA in a field setting, we applied the assay to single-celled isolates collected from an ocean observatory.

#### Miniaturisation of Culturing and Trait Measurements

The validation of the QPA was conducted on six strains of *Thalassiosira* spp. (CCMP1010, CCMP1050, CCMP1059, CCMP1587, CCMP2929, and CCMP3367) grown in 12-well tissue culture plates (2 plates, *n* = 3 per plate, randomly assigned well position, culture volume = 4.4 mL) under “standard” conditions (experiments performed in October 2020). Specifically, cultures were grown in artificial seawater with f/2 nutrients (Guillard, 1975), incubated at 20°C with irradiance of 60 μmol photons m^−2^s^−1^ (12:12 h). Two independent plates were used to observe the extent of plate effects on trait values, with biological replicates randomly positioned within each plate. Principal component analysis was used to visualise the strains' multivariate phenotypes in a 2D trait-scape (described below).

To assess phenotypic differences that may be brought about by culturing in well plates, cultures were also grown in 50 mL tissue culture flasks (*n* = 3 per strain) under the same “standard” conditions described above. Univariate ANOVAs (or equivalent non-parametric methods) were used to test significant differences in individual trait values between the flasks and the two independent well plates for each strain. Pairwise PERMANOVA was used to determine differences in multivariate phenotypes between vessel (flasks and each well plate) for each strain. Multivariate trait datasets were analysed using principal component analysis and by generating correlation matrices of the individual trait values. To compare the positioning of the strains within the two PCA plots, Euclidean distances between the strain centroids (multivariate means) were calculated for each plot, followed by a linear regression to assess their correspondence. We also performed PCA on standardised correlation matrices which included trait data from both flasks and plates, excluding and including vessel type as an additional variable. This allowed us to test whether additional variance was explained by vessel type. The QPA was also assessed through permutational analysis of variance (PERMANOVA) to test the factors of strain and vessel type and determine whether culturing in plates (“miniaturization”) had any influence on overall phenotypes.

#### Determination of Trait Values for Four Strains Across 36 Environments Using the QPA

To assess the utility of the QPA for assessing phenotypic plasticity in many environments, four strains (CCMP1093, CCMP2929, CCMP3264, and CCMP3367) were grown in 24-well plates (culture volume = 2.2 mL) in 36 different environments. There were two biological replicates for strains CCMP1093 and CCMP2929, and four biological replicates for strains CCMP3264 and CCMP3367. These environments were created using a fully factorial design of 6 different nutrient regimes at two different irradiances 60 or 200 μmol photons m^−2^s^−1^ at three different temperatures: 14, 20, and 28°C. The nutrient conditions were: f/2 nutrients in artificial seawater, modified f/2 nutrients in artificial seawater with either elevated DIC (2 mg/L), or low iron (0.05 μM compared to 11.7 μM in f/2), low nitrate (4 μM compared to 882 μM in f/2), low phosphate (0.2 μM compared to 36.2 μM in f/2), or low salinity (15 ppt compared to 35 ppt in normal artificial seawater). Low light conditions were achieved by a neutral density filter (Lee Filters, UK) covering the lid of each, resulting in an incident light level of 60 μmol photons m^−2^s^−1^.

Using 24-well plates for multi environment studies increases through-put for *in vivo* Chl-a plate-reading and reduces the shelf area required in culture incubators. However, this reduces each culture volume to 2.2 mL which reduces the number of traits that can be measured from each experimental unit. There are also considerations of handling and analysis time for larger numbers of samples. Here we chose four flow cytometry traits to demonstrate the utility of a reduced-trait QPA to evaluate phenotypic plasticity: population growth rate, cell size, granularity, and Chl-a content.

#### Using the QPA on Newly Isolated Diatoms

To demonstrate the utility of the QPA as a method for phenotyping fresh isolates that represent the minimal viable population for the QPA, five single-celled centric diatoms were isolated on 1 September 2020 from the Port Hacking National Reference Station, a time-series observatory in Australia's Integrated Marine Observing System (IMOS). Isolates were maintained at 18°C with a 12:12 light cycle (60–100 μmol m^−2^ s^−1^) in 24 well tissue culture plates (culture volume = 2.2 ml) in filtered natural seawater (NSW) from the collection site enriched with f/20 nutrients (Guillard, 1975). Cells were transferred into fresh sterile media every 2–3 days. Once 200–300 cells were established, half of the new culture was transferred into natural seawater from Port Hacking with f/20 nutrients and maintained in the same culturing conditions (NSW), and half was transferred into f/2 nutrients in artificial seawater (ASW), the long-term culturing conditions of other strains used in this study. After 2 weeks of maintenance under these conditions, experimental cultures were set up in 12-well tissue culture plates to phenotype the strains in both media conditions (*n* = 3 per media condition per strain). In the QPA, some isolates grew only for 3–4 days before reaching stationary phase, which was unexpected based on the growth dynamics of the domesticated *Thalassiosira* strains (Supplementary Figure 2). As silicification was a trait requiring 24 h of exponential growth, we were unable to accurately quantify this trait after “missing” the window of growth. All other traits were measured and analysed using PCA. PERMANOVA was used to assess differences between phenotypes of strains grown in the two different culture media.

### Data Analyses and Statistical Tools

Data processing from the plate reader, flow cytometer, and WATER PAM, as well as descriptive statistics were done in Microsoft Excel. Basic statistics (ANOVAs, Kruskall-Wallace tests, linear regressions) were performed in R (R Core Team, 2021). The phenotypes derived from the QPA were assessed in two ways: (1) using PCA which allows for the identification of trends in multivariate space, and (2) using pair-wise trait correlations. Principal component analyses were conducted using the Vegan package (Oksanen et al., 2019) in R. Trait data was standardised before all PCA analysis to account for differences in the scale of trait measurements. Distances between PCA co-ordinates were calculated using the equation:


$$\begin{array}{l} \text{Distance}=\sqrt{\frac{{\left(y2-y1\right)}^{2}}{{\left(x2-x1\right)}^{2}}} \end{array}$$

Where x and y are the PCA coordinates of the centroids of each strain/temperature combination. The direction of the distance change was calculated using:


$$\begin{array}{l} \text{Direction }\left(\text{angle}\right)=\text{ta}{\text{n}}^{-1}\frac{y2-y1}{x2-x1} \end{array}$$

Pairwise PERMANOVA was conducted using the pairwise.adonis package implemented in R (Martinez Arbizu, 2020). Trait correlation matrices were generated using the corrplot package (Wei and Simko, 2017), also in R.

## Results

### Miniaturised Culturing and Trait Measurements Using the QPA

**Figure 2:**
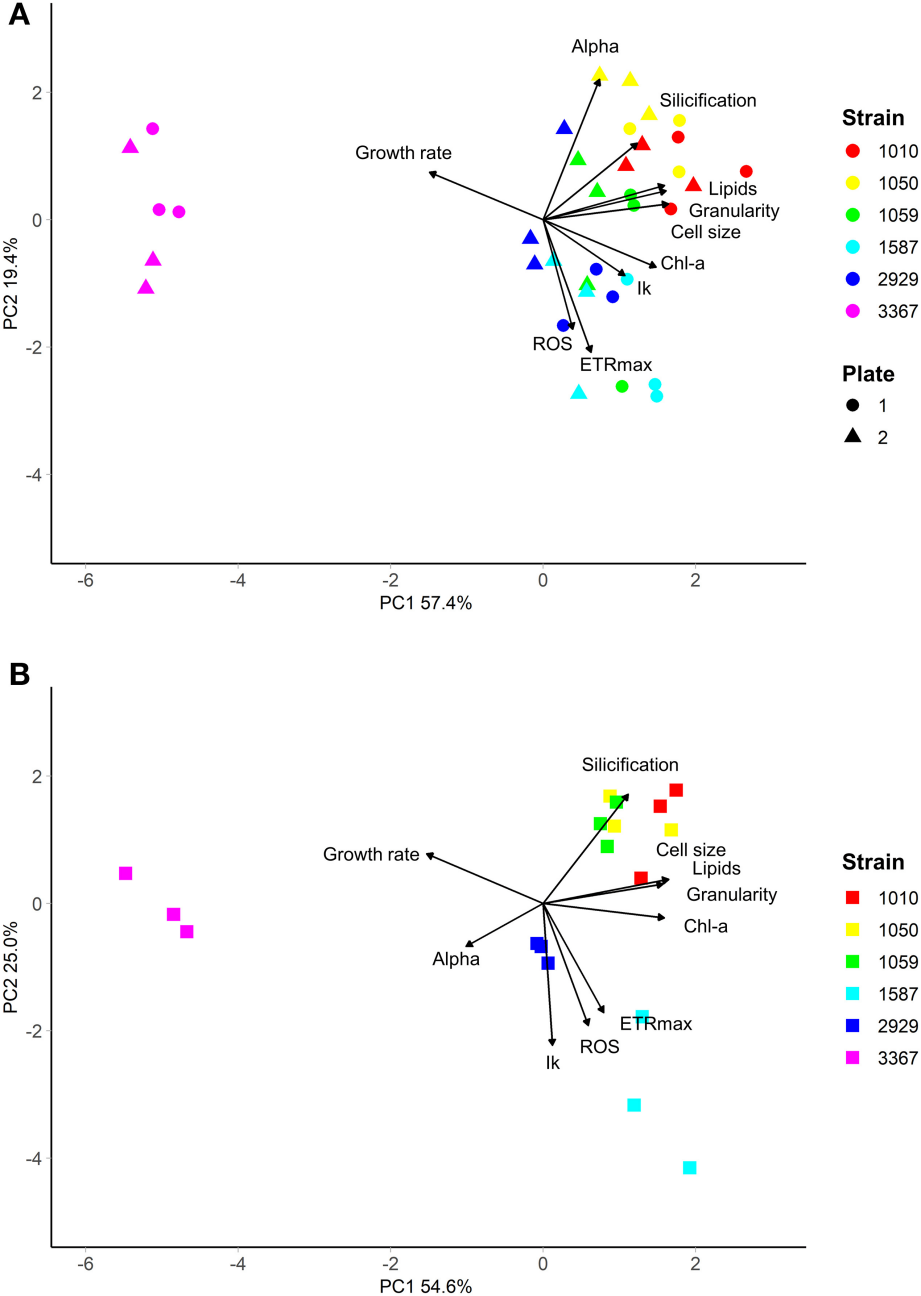
Multivariate phenotypes of six *Thalassiosira* strains grown in either **(A)** multiwell plates (*n* = 6 per strain, split across two independent plates), or **(B)** 50 mL tissue culture flasks (*n* = 3 per strain), visualised using Principal Component Analysis. Strains were grown in in artificial seawater (ASW) with f/2 media (Guillard, 1975) at 20°C, with 60 μmolm^−2^s^−1^ of light on a 12:12 light cycle. Strains are shown in different colours.

**Figure 3:**
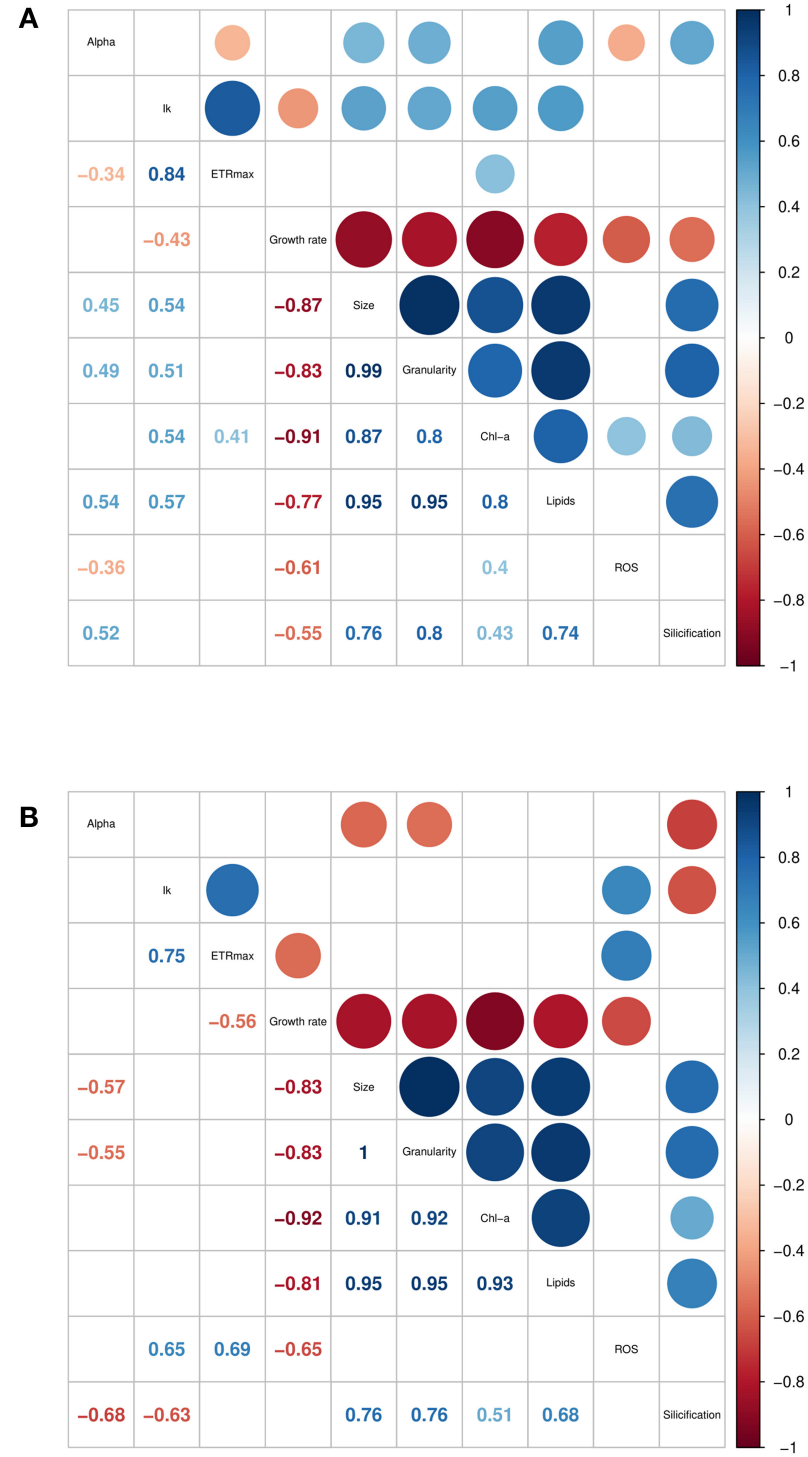
Correlation matrices of trait data collected for six *Thalassiosira* strains, grown in **(A)** multiwell plates, and **(B)** 50 mL tissue culture flasks. Only significant (*p* < 0.05) correlation coefficients are shown. Trait units are found in Supplementary Table 1.

The traits incorporated into the QPA were based on published methods (Ralph and Gademann, 2005; Knauert and Knauer, 2008; Pereira et al., 2011; Rumin et al., 2015; Baker et al., 2016). The QPA integrates multiple traits into a single workflow, thereby providing a more holistic view of microalgae compared to traditional approaches, allowing the identification of strains with similar or divergent phenotypes. An example of the PCA or “trait-scape” for *Thalassiosira* grown in multi-well plates under “standard conditions” is shown in Figure 2A and the corresponding correlation matrix for the trait-scape is given in Figure 3A. In *Thalassiosira*, cell size is significantly positively correlated with Chl-a and lipid content, granularity and silicification (Figure 3A), and negatively correlated with growth, consistent with it being a “master trait” (Litchman and Klausmeier, 2008). However, photophysiological traits (alpha, I_k_) were marginally correlated with ETRmax, and ROS was uncorrelated with cell size (Figure 3A).

#### Plate Effects

Between the two independent well plates, there was generally no difference in observed trait values, indicating minimal plate effects (Table 2). The exceptions were strains CCMP2929 and CCMP3367, for which lipid content per cell varied between the plates (14 and 19% lower in plate 2, respectively: Tukey HSD *p* < 0.05). For strain CCMP2929, I_k_ and ETRmax were also different between plates (6 and 9% decrease from plate 1 to 2, respectively: Tukey HSD *p* < 0.05), and for strain CCMP3367 there was a 3% decrease in Chl-a between plate 1 and 2: Tukey HSD *p* < 0.05. While we did not test the source of inter-plate variability extensively (e.g., incubator heterogeneity), we used random positioning to account for intra-plate variation and found low overall trait value variation using this design.

**Table 2:** Summary of comparisons of individual trait values and integrated phenotypes between flask and plate-grown cultures for six *Thalassiosira* strains.

	**Flask and plate difference**						**Between plate difference**					
Trait|Strain	CCMP1010	CCMP1050	CCMP1059	CCMP1587	CCMP2929	CCMP3367	CCMP1010	CCMP1050	CCMP1059	CCMP1587	CCMP2929	CCMP3367
Growth	^**^	^***^	^***^	^**^	^**^	^***^	-	-	-	^**^	-	-
Size	-	-	-	-	-	^***^	-	-	-	-	-	-
Granularity	-	-	**F-1	**F-1	-	-	-	-	-	-	-	-
Chl-a	-	KW -	KW -	-	-	-	-	KW-	KW-	-	-	^**^
Lipid content	^**^	^***^	^***^	-	F-1 ^***^	F-1^***^	-	-	-	-	^**^	^**^
ROS	-	-	-	^**^	-	^**^	-	-	-	-	-	-
Alpha	^***^	^***^	^**^	^**^	-	-	-	-	-	-	-	-
I_k_	^**^	^***^	^***^	-	^***^	^**^	-	-	-	-	^**^	-
ETRmax	F-1 ^**^	-	F-1^**^	-	F-1^**^	-	-	-	-	-	^**^	-
Integrated phenotype	-	-	-	-	-	-	-	-	-	-	-	-

*For individual traits, results represent post-hoc Tukey's HSD test following univariate ANOVAs*

*** *significant difference P < 0.005*,

** *significant difference P < 0.05. “-” no significant difference. KW indicates Kruskall-Wallace test with Dunn's post-hoc comparisons, used when ANOVA assumptions were not met. F-1 indicates a difference between flasks and one growth plate but not the other. For integrated phenotypes, results are from pairwise perm PERMANOVAs comparing flasks to each growth plate. “-” indicates no significant difference (p > 0.05)*.

#### Effects of Miniaturised Culturing on Trait Values and Phenotypes

Some trait values varied between the cultures grown in 50 mL flasks vs. multi-well plate (Table 2). Population growth rates were consistently lower in the flasks for all strains (Tukey HSD *p* < 0.05), and at least one of the photophysiological traits differed between the vessels for all strains (Table 2). Lipid content was also significantly higher in the plate-grown cultures (Table 2). When assessed using multivariate methods (pairwise PERMANOVA) however, there were no significant differences in integrated phenotype between the flasks and either growth plates, or between the plates themselves (Table 2).The 2D trait-scape generated from cultures grown in flasks was similar to the trait-scape for plate-grown cultures (Figure 2B). Euclidean distances between the centroids (multivariate mean phenotype) of each strain were positively correlated between the two culturing methods (*R*^2^ = 0.93, *p* < 0.05), demonstrating that the strain-strain relationships were preserved between the small volume multi-well plates and larger volume flasks. The underlying components of the trait-scapes showed similar patterns, i.e. the trait correlations (Figure 3B) and the contributions of the traits to the PC axes (Supplementary Table 2).

When vessel type was included as an additional variable in the PCA, a similar amount of variance in phenotypes was explained by the first two principal components (73.13 vs. 73.86%; Supplementary Figure 3), demonstrating that vessel type is redundant when considering the outcome of the QPA workflow. Furthermore, the PERMANOVA showed that inter-strain differences had a substantially greater impact on the phenotype than vessel type (PERMANOVA: *F* = 1283.714 vs. *F* = 49.354, respectively). Phenotypes were distinct between strains (*p* = 0.001) yet the interaction between strain and vessel type was insignificant (*p* = 0.435), indicating that vessel type does not impact the ability to distinguish strains using the QPA. In summary, individual traits may differ between flasks and plates, but their relationship to one another and how they combine to form the phenotype is equivalent among vessel types.

### Determination of Phenotypic Plasticity Using the QPA

Out of the 432 culture wells inoculated into different environments, 364 grew successfully and traits could be quantified. Cultures that did not grow (i.e., showed no increase in *in vivo* Chl-a fluorescence over time) or crashed before phenotyping was possible, were excluded from the analyses. While variable amongst strains, the environments that were most commonly restrictive for growth were the low nitrogen and phosphorus conditions.

**Figure 4:**
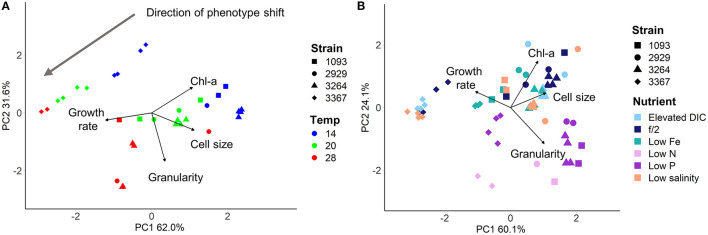
Four *Thalassiosira* strains grown under differing culture conditions, visualised using Principal Component Analysis. **(A)** Strains grown in f/2 media under 200 μmol m^−2^ s^−1^ light in three different temperatures (colours - showing unidirectional shift in the trait scape). **(B)** Strains grown at 20°C with 200 μmol m^−2^ s^−1^ light in six different nutrient environments (colours). Directional shifts in phenotype can be seen in the low nitrogen and low phosphorus treatments.

Adjustment of the multivariate phenotype under different environmental conditions was assessed by visualising the direction of “movement” of strains within the 2D trait-scape. For example, when visualising a subset of the data (Figure 4A), a similar direction of phenotypic adjustment was observed for all four strains in the three different temperatures, corresponding to an increase in growth rate and decrease in cell size and Chl-a content with increasing temperature (Figure 4A; Table 3). The magnitude of change ranged from 1.11–1.90 (diagonal distance in the PCA) for 14–20°C and 0.92–2.03 for 20–28°C shift (Table 3). The direction of the shift was between 11.9 and 36° (from the horizontal) between 14 and 20°C and 15 and 86.2° between 20 and 28°C (Table 3). These data demonstrate the greater variability in response regarding both magnitude and direction of phenotypic shift when temperature shifted upwards to 28°C from 20°C compared to when it shifted downwards by 6°C from 20°C (Table 3). The relationship between temperature and growth rate was linear for all strains, with CCMP3367 having the highest rate of change (0.0605 d^−1^ per °C; Table 3).Under different nutrient or media conditions, there was a shift in the phenotype under low nitrogen and phosphorus conditions associated with increased cell size and granularity accompanied by reduced growth rates. The other conditions did not show consistent patterns of change within the trait-scape (Figure 4B).

**Table 3:** Distance and direction (degrees from the horizontal) between phenotype centroids for strains grown at 14, 20, and 28°C in f/2 media at 200 μmol m^−2^s^−1^ of light on a 12:12 light cycle.

**Strain**	**Distance 14–20^**°**^**	**Direction14–20^**°**^**	**Distance 20–28^**°**^**	**Direction20–28^**°**^**	**Slope of growth rate vs. temperature**
CCMP1093	1.51	25.2	1.39	15.0	0.0592
CCMP2929	1.11	16.9	1.43	86.2	0.0289
CCMP3264	1.61	11.9	2.03	47.6	0.0571
CCMP3367	1.90	36.0	0.92	39.6	0.0605

*Slope of temperature vs. growth rate across the three temperatures is also shown for each strain*.

### Trait Quantification of Newly Established Cultures From Isolates

**Figure 5:**
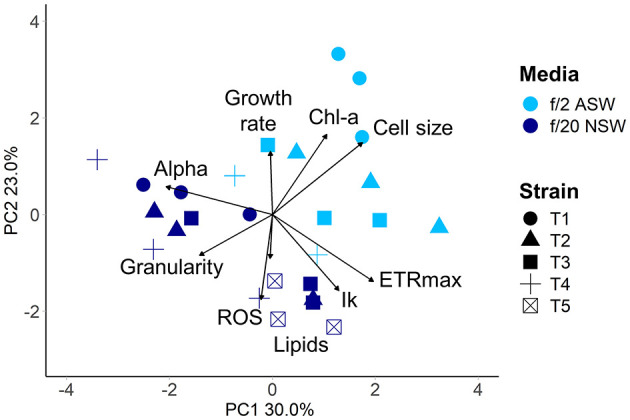
Multivariate phenotypes of newly isolated centric diatom strains from Port Hacking IMOS National Reference Station visualised using Principal Component Analysis. Strains were grown in either artificial seawater with f/2 nutrients (light blue), or natural seawater with f/20 nutrients (dark blue) at 18°C with a 12:12 light cycle (60–100 μmol m^−2^ s^−1^). Strains are indicated by symbol. Strain T5 did not grow in artificial seawater.

Within 4 weeks of isolation, the five new diatom isolates had grown to sufficient cell densities to perform the QPA. This equates to enough cells to inoculate three replicate 4.4 mL wells with 2,000 cells mL^−1^, or a total of 26,400 cells. Four out of the five isolates grew in both media (ASW with f/2 and NSW with f/20), with the fifth growing only in the NSW treatment. The isolates had similar individual trait values when grown in the same media (Supplementary Table 3), which may be due to them being from the same population from the same diatom spring bloom. However, phenotypes were divergent in the two media types (PERMANOVA pseudo *F* = 10.19, *P* = 0.005, Figure 5), demonstrating that in the early phase of their establishment, strains were highly responsive to their different culture environments. Phenotypes were also more variable in the artificial seawater environment with f/2 media, with one strain being unable to grow (Figure 5).

## Discussion

The QPA expands the current scope of phenotyping microalgae by combining 10 trait quantification methods into a single high-throughput well-plate-based format. Due to constraints concerning time, space or resources, previous phenotyping of microalgae largely focused on either a few traits in multiple taxa/environments or multiple traits in few taxa/environments. The QPA overcomes this limitation, enabling multiple strains or species to be grown in 36 or more environments (up to 432 wells) and for traits to be measured by an individual researcher within a reasonable timeframe, with standard equipment and realistic laboratory workflows. Trait values are measured on the same experimental unit, which is advantageous both biologically and statistically.

A challenging aspect of understanding the performance of microalgae in diverse environments or under multi-dimensional global change is deciding the number of traits to measure and determining which traits can help predict intra- and inter-functional group dynamics. Here we have shown that the QPA enables investigations that yield more holistic information about microalgal phenotypes and allows comparison of distinct responses to environmental conditions that address finer-scale diversity shifts within the diatom functional group. The use of flow cytometer calibration beads, negative unstained controls, and the normalisation of traits to take cell size or growth into account, provides a robust methodology for quantifying traits reliably. Given the biomass needed for quantifying all 10 traits, the limit of detection is an initial 2,000 cells mL^−1^, or a total of 26,400 cells.

**Figure 6:**
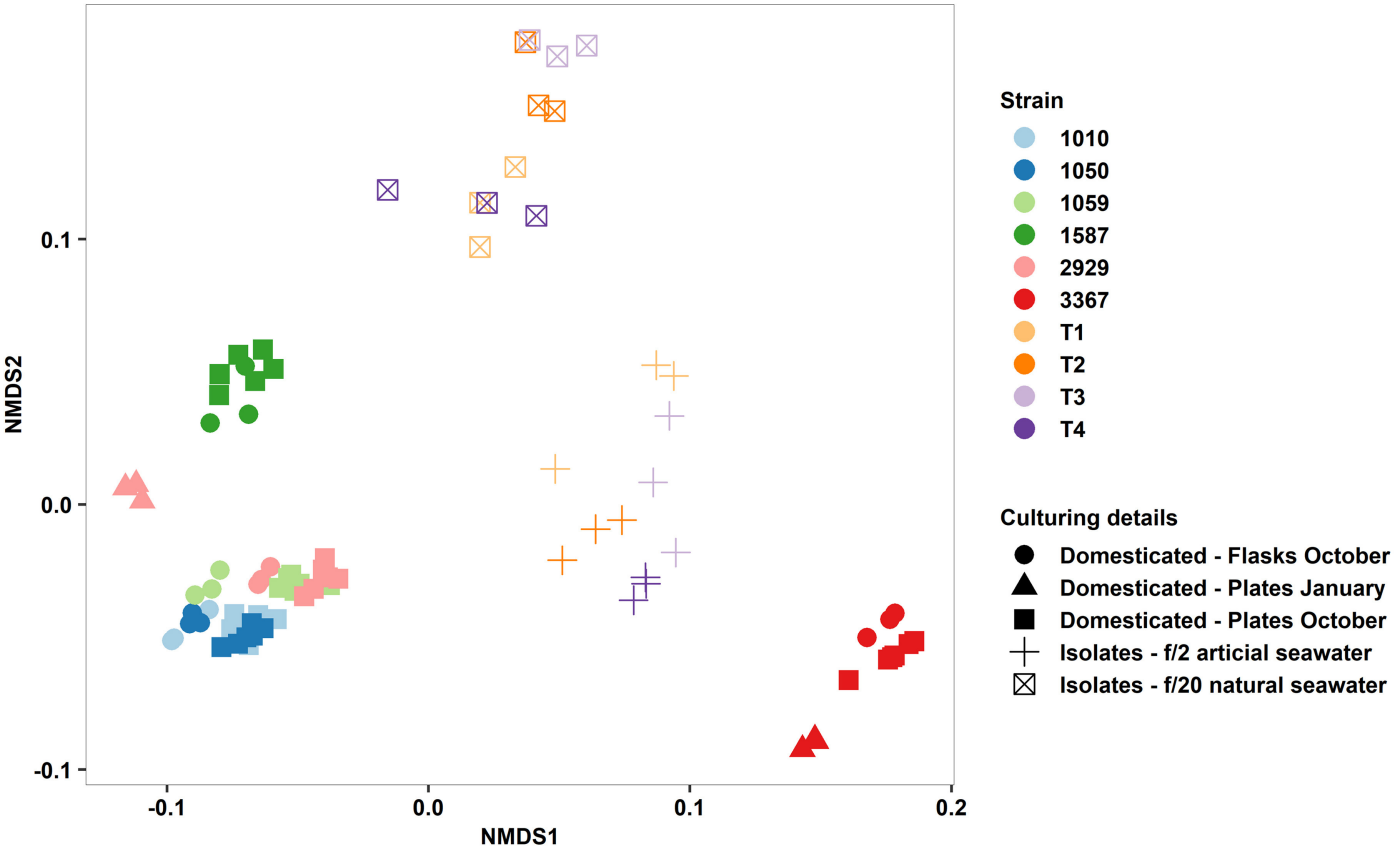
Non-metric multidimensional scaling plot of multi-trait phenotypes of domesticated *Thalassiosira* strains (CCMP1010, CCMP1050, CCMP1059, CCMP1587, CCMP2929, and CCMP3367) and newly established isolates from Port Hacking, Sydney, Australia (T1–4). Input traits were: Growth rate, cell size, granularity, Chl-A, Lipid content, ROS content, alpha, ETRmax, and I_k_. Symbols represent type of strain, culture vessel, time of phenotyping, and culture media.

In terms of reproducibility of the QPA, we tested the factors influencing phenotypic variance within and between our diatom strains, observing their relative magnitude. Vessel type (multi-well plate vs. culture flask) had a minimal effect on strain phenotype as did time (traits quantified in January vs. October, tested for two strains), but there was a considerable effect of culture medium (Figure 6). Phenotypic divergence amongst these isolates could be attributed to seawater source (natural vs. artificial) and nutrient concentration (f/20 vs. f/2), the latter of which was also shown to induce phenotypic plasticity in established strains (Figure 4), but could also be due to the more variable nature of fresh isolates compared to domesticated strains. Determining whether the magnitude of phenotypic variance differs between fresh isolates and domesticated strains is an area of further research. To facilitate comparisons across future studies, we recommend that researchers publish raw trait values and consider the use of reference strains as an “internal standard” to ensure measurements made within their own laboratories are reproducible. Also, if cross-laboratory comparisons are to be made, standardising media and seawater preparation is essential.

Through miniaturisation of culture volumes, the QPA opens up the possibility of phenotyping fresh isolates from experimental enclosures or field sites, when cells are potentially more representative of their *in situ* phenotype. High-throughput multivariate phenotyping also provides more nuanced information about the growth strategies of microalgae, as well as observing phenotypic plasticity, trait correlations, and multi-trait trade-offs.

Trade-offs are often generally assumed to occur between pairs of traits, such as growth rate and cell size (Finkel et al., 2010), however there is evidence that trade-offs can occur between three or more traits (Edwards et al., 2011). In our study, we show a consistent shift in phenotype with a change in temperature across four strains (Figure 4A). This shift in multivariate space captures the up to four-dimensional trade-offs that may be occurring between the four measured PCA input traits, without having to plot or examine them individually.

These data also demonstrate that the trait-scape for microalgae has underlying constraints, governed by the relationships and trade-offs between phenotypic traits. These constraints influence the ways in which diatoms can respond to stressors, and the growth strategies that they can utilise in different environments (Argyle et al., in press). By exposing multiple strains to multiple environments in large comprehensive experiments using the QPA, we can push microalgae to their physiological limits and gain insight into their phenotypic constraints and viable trait combinations. Multi-stressor experiments done using the QPA may result in novel phenotypes, representing diatoms at the margin of viability. Such experiments can be used to define the limits of diatom plasticity, an important consideration when assessing their ability to evolve (Pfennig et al., 2010; Malcom et al., 2015; Schaum et al., 2018). Trait-plasticity is rarely included in biogeochemical models, however it is highly relevant given the high phenotypic diversity known in the phytoplankton and more specifically in diatoms (Godhe and Rynearson, 2017). The QPA therefore provides a tool to estimate trait variation and relationships that could be used to inform trait-based models of microbial evolution (Walworth et al., 2021).

During the development of the QPA, it became clear that phenotyping from small volume cultures is dependent on sufficient growth of the algal cells, and that as environments become more stressful, phenotyping becomes more challenging. This is not only because growth is reduced and the availability of cells or biomass for trait measurements becomes limited, it is also because of the tendency for cultures to crash, leaving insufficient time to measure traits. In these instances, pilot trials of growth can be used to determine viable environments before investing in full-resolution trait measurements. Such “growth-only” experiments can also be used to choose environments for experiments and estimate the limits and optima of growth or tolerances to parameters of interest (e.g., temperature, salinity, or nutrient levels) in a high-throughput manner. Multi-environment traits such as temperature optima and half-saturation constants for nutrient uptake (Ks) are valuable parameters frequently used for modelling purposes (Edwards et al., 2012). During this study we examined whether half-saturation (Ks) could be measured using multi-well plates. We observed a Michaelis-Menten type response of growth under different nitrate concentrations (Supplementary Figure 4), as would be expected, but at low nutrient concentrations approaching environmentally-realistic ocean levels, limited microalgal biomass made phenotyping challenging. To assess the cells in a nutrient limited state, it may be necessary to acclimate the cells in larger volumes prior to undertaking the QPA. Finally, we have not addressed the phenomenon of cells which may persist but not grow during an experiment. Cell viability is verified within the QPA through determination of active Chl-a fluorescence. For diatoms, the ability to persist in suboptimal conditions is part of their overall survival strategy (Smayda and Mitchell-Innes, 1974; Kato et al., 2004). Cells can be phenotyped even when cell division has ceased, however we advise careful consideration of these results as they represent cells that are in a different physiological state compared to an actively growing culture.

Through development of a multi-trait assay, we have demonstrated that the integrated phenotype derived from the QPA workflow is less sensitive to vessel type than individual traits, overcoming a limitation of traditional phenotyping approaches. The QPA methodology therefore facilitates comparisons amongst phenotypes and highlights that comparison of individual trait values between different studies should consider how cells were cultivated. The QPA has been designed as a framework for the inclusion of other traits and taxa depending on the needs of the researcher. Not only is the QPA applicable to a range of diatoms, including taxa with pennate morphology and larger cell sizes (depending on flow cytometer aperture), it has the potential to accommodate other taxonomic lineages such as coccolithophores and dinoflagellates. These functional groups are largely unicellular with suitable size and morphology to grow in multiwell plates. The silicification assay would best be substituted for another trait given that the latter functional groups have no requirement for silica, but any new trait would require optimisation e.g., determining the appropriate stain concentration and time.

## Conclusion

The QPA presents a novel and flexible approach to high-throughput phenotyping for both industrial and ecological applications. The lack of searchable trait databases has been identified as a key limitation in the field of algal phenomics (Fabris et al., 2020). The method described here will facilitate the rapid collection of high-dimensional phenotypic data for microalgal taxa that could be included in databases as they become available. The utility of this assay both in the laboratory and in the field creates opportunities for rapid phenotyping that has been previously limited. This method also creates a framework for a standardised approach to phenotyping that could be used to create cross-comparable datasets.

## Data Availability Statement

The raw data supporting the conclusions of this article will be made available by the authors, without undue reservation.

## Author Contributions

NL, SC, and MD: conceived the study. PA, JH, MD, SC, and NL: designed the experiments. PA and JH: conducted the experiments and analysed the data. All authors wrote the paper.

## Funding

This research was supported by the Gordon and Betty Moore Foundation Marine Microbes Initiative (MMI 7397 to NL, MD, and SC).

## Conflict of Interest

The authors declare that the research was conducted in the absence of any commercial or financial relationships that could be construed as a potential conflict of interest.

## Publisher's Note

All claims expressed in this article are solely those of the authors and do not necessarily represent those of their affiliated organizations, or those of the publisher, the editors and the reviewers. Any product that may be evaluated in this article, or claim that may be made by its manufacturer, is not guaranteed or endorsed by the publisher.
