# Supplementary material for: A High-Throughput Assay for Quantifying Phenotypic Traits of Microalgae
Source: Front Microbiol. 2021 Oct 6;12:706235. doi: 10.3389/fmicb.2021.706235 (PMC8528002; doi:10.3389/fmicb.2021.706235)
Supplement: Supplementary file 1 [file Data_Sheet_1.pdf]

Supplementary Table S1. Details of traits included in the Quantitative Phenotyping Assay (QPA) including details considered for each method and optimisation.

Trait	Definition	Method of measurements	Unit of measurement in the QPA	Per cell or population level	Volume	Live or fixed cells	Notes and limitations	Things requiring optimisation for each taxa	Method descriptions and example use
**Growth Rate**	Change in cell concentration over time	*In vivo* fluorescence measured over multiple time points using a plate reader. Can be validated using flow cytometry counts.	divisions day^{-1}	population level	2-10 mL growth culture depending on size of well-plate chosen	Live		Initial inoculum concentration. Gain sensitivity of plate reader.	Wood et al. 2005
**Cell Size**	Size of an individual cell	Flow cytometry, comparison to standard size beads	µm (equivalent diameter)	per cell (size corrected)			Gives an indication of relative size based on a sphere. Error may vary with cell shape	Size range of reference beads, fixative concentration	Hyka et al. 2013, Schaum et al. 2016
**Cell complexity or granularity**	Cell complexity or granularity	Flow cytometry, comparison to standard size beads	RFU per cell (size corrected)	per cell (size corrected)	200 µL fixed with 20 µL paraformaldehyde	Fixed		Fixative concentration	Hyka et al. 2013
**Chlorophyll pigment concentration**	Concentration of chlorophyll per cell	Flow cytometry, comparison to fluorescent standard bead	RFU per cell (size corrected)	per cell (size corrected)			Fluorescence degrades after fixation and/or storage	Fixative concentration	Hyka et al. 2013, Schaum et al. 2016
**Neutral Lipid content**	Concentration of neutral lipids present in each cell	Flow cytometry, BODIPY stain	RFU per cell (size corrected)	per cell (size corrected)				Stain concentration and incubation time	Rumin et al. 2015
**Silicification of frustule**	Proxy of silica uptake by the cells over 24 hours	24-hour incubation with PDMPO stain followed by flow cytometry	RFU per cell (size corrected)	per cell (size corrected)	500 µL treatment + 500 µL control	Live	PDMPO uptake indicative of overall silica uptake (frustule + vacuoles)	NB Only applicable for silicifying taxa	Leblanc and Hutchins 2005, McNair et al. 2015
**Reactive oxygen species content**	Concentration of reactive oxygen species present in each cell	H_2DCFDA incubation, fluorescence read using a plate reader	RFU per cell (size corrected)	per cell (size corrected)	500 µL treatment + 500 µL control	Live	Incubation occurs in the dark therefore light treatments are not accounted for	Stain concentration and incubation time	Knauert and Knauer 2008
**<u>Photophysiology traits</u>**									
**ETRmax**	Maximum electron transport rate		µmol electrons m−s−1						
**Ik**	Irrandiance at half of ETRmax	PAM rapid light curve	µmol photons m^{-2}s^{-1}	population level	1 mL	Live		Cell concentration at time of mesurement	Ralph and Gademann 2005
**alpha**	Photosynthetic efficiency under light limitation		mg C(mg Chl a)$^{-1}$ h^{-1}(µmol photons m^{-2}s^{-1})$^{-1}$						

Trait table references

Hyka P, Lickova S, Přibyl P, Melzoch K, Kovar KJBa. 2013. Flow cytometry for the development of biotechnological processes with microalgae. *Biotechnology Advances.* 31:2-16.

Knauert S, Knauer K. 2008. The role of reactive oxygen species in copper toxicity to two freshwater green algae *Journal of Phycology.* 44:311-319.

Leblanc K, Hutchins DA. 2005. New applications of a biogenic silica deposition fluorophore in the study of oceanic diatoms. *Limnology and Oceanography: Methods.* 3:462-476.

McNair HM, Brzezinski MA, Krause JW. 2015. Quantifying diatom silicification with the fluorescent dye, PDMPO. *Limnology and Oceanography: Methods.* 13:587-599.

Ralph PJ, Gademann R. 2005. Rapid light curves: a powerful tool to assess photosynthetic activity. *Aquatic Botany.* 82:222-237.

Rumin J, Bonnefond H, Saint-Jean B, Rouxel C, Sciandra A, Bernard O, Cadoret J-P, Bougaran G. 2015. The use of fluorescent Nile red and BODIPY for lipid measurement in microalgae. *Biotechnology for Biofuels.* 8:42.

Schaum C-E, Rost B, Collins S. 2016. Environmental stability affects phenotypic evolution in a globally distributed marine picoplankton. *The ISME Journal.* 10:75-84.

Wood AM, Everroad R, Wingard L. 2005. Measuring growth rates in microalgal cultures. Pages 269-288. *Microalgae Culturing Techniques*, vol. 18.

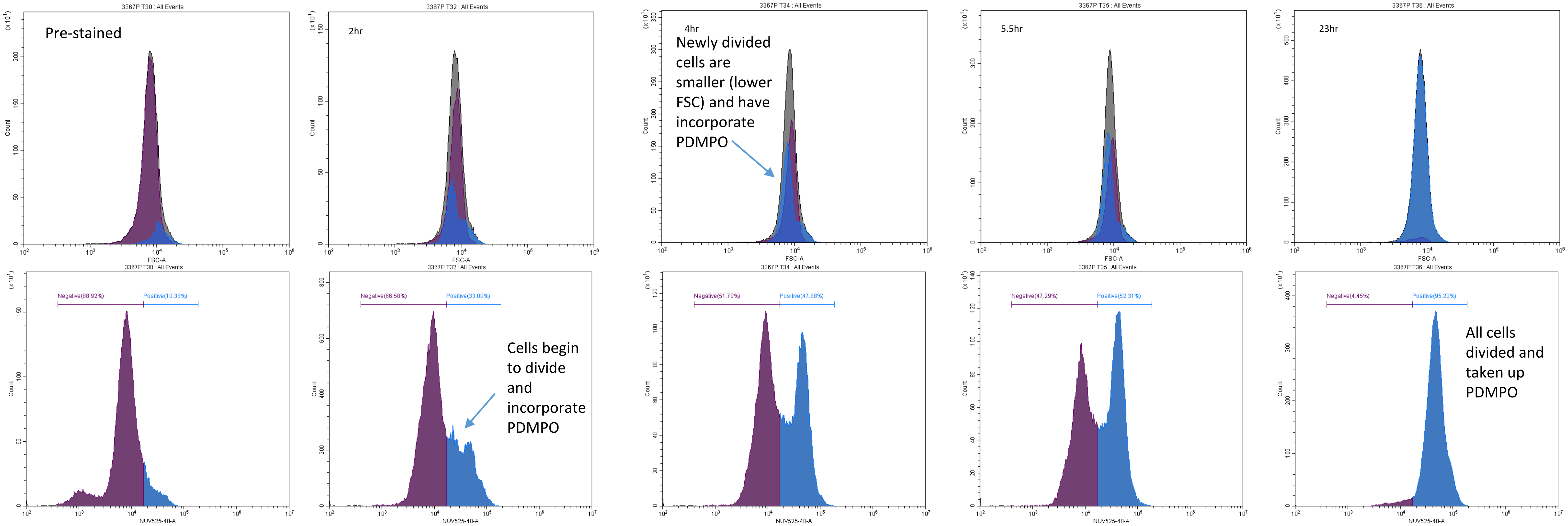

Supplementary Figure S1. Time series of PDMPO uptake by strain 3367 (*Thalassiosira pseudonana*) over time. Plots are histograms of signal intensity for the Forward scatter (proxy for size) or NUV525-40A (PDMPO) detectors. At time zero there is auto-fluorescence by the cells detected by the in the NUV525-40A. Following staining the signal increases over time as PDMPO is incorporated into the cells. The double peak of PDMPO fluorescence represents newly divided cells that are smaller (as determined by side scatter) but have higher PMDPO as it is incorporated through cell division. After 23 hours all cells have divided and incorporated PDMPO.

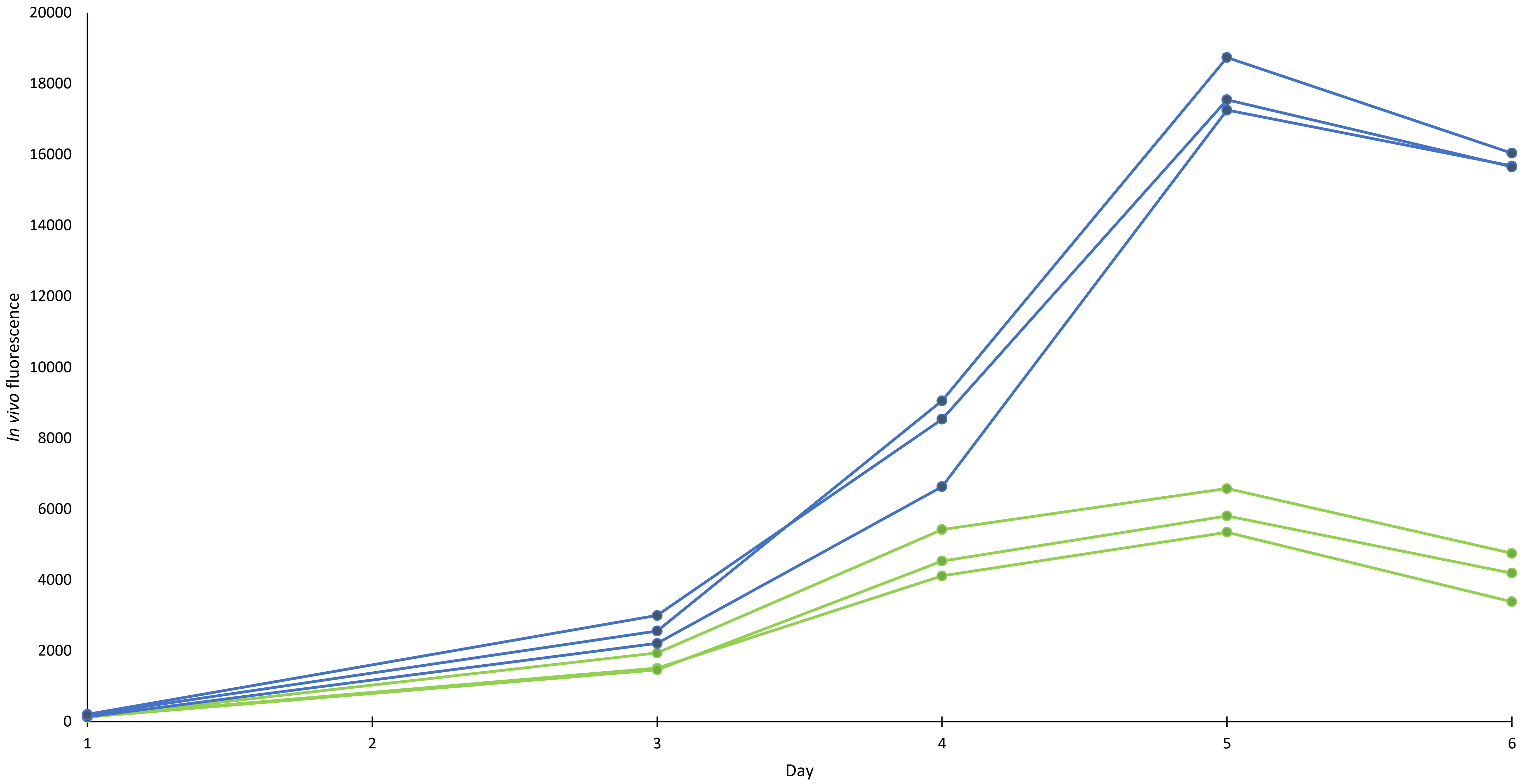

Supplementary Figure S2. *In vivo* fluorescence over time for the newly isolated strain T1 grown in artificial seawater with f/2 media (green) and f/20 media with natural seawater (blue). Each line represents a biological replicate, n=3 per treatment.

Supplementary Table T2. Trait contributions to (A) and correlations with (B) the first two PC axes for trait-scapes generated using trait values from *Thalassiosira* strains grown in tissue culture flasks and plates.

**A**	**PC1**		**PC2**	
**Contributions of traits to PC axes**	Plates	Flasks	Plates	Flasks
**Alpha**	3.41	6.40	30.34	2.83
**Ik**	7.16	0.09	4.78	31.04
**ETRmax**	2.46	3.93	27.04	18.37
**Growth rate**	13.85	14.55	3.45	3.79
**Cell size**	16.89	16.93	0.38	0.89
**Granularity**	16.26	16.89	1.30	0.91
**Chl a**	13.68	15.77	3.45	0.32
**Lipids**	15.81	15.44	1.81	0.58
**ROS**	0.94	2.20	18.43	22.92
**Silicification**	9.55	7.79	9.02	18.34

**B**	**PC1**		**PC2**	
**Correlations of traits to the PC axes**	Plates	Flasks	Plates	Flasks
**Alpha**	0.44	-0.60	0.77	-0.29
**Ik**	0.64	0.07	-0.31	-0.94
**ETRmax**	0.38	0.47	-0.73	-0.73
**Growth rate**	-0.89	-0.91	0.26	0.33
**Cell size**	0.98	0.98	0.09	0.16
**Granularity**	0.97	0.98	0.16	0.16
**Chl a**	0.89	0.94	-0.26	-0.10
**Lipids**	0.95	0.93	0.19	0.13
**ROS**	0.23	0.35	-0.60	-0.81
**Silicification**	0.74	0.66	0.42	0.73

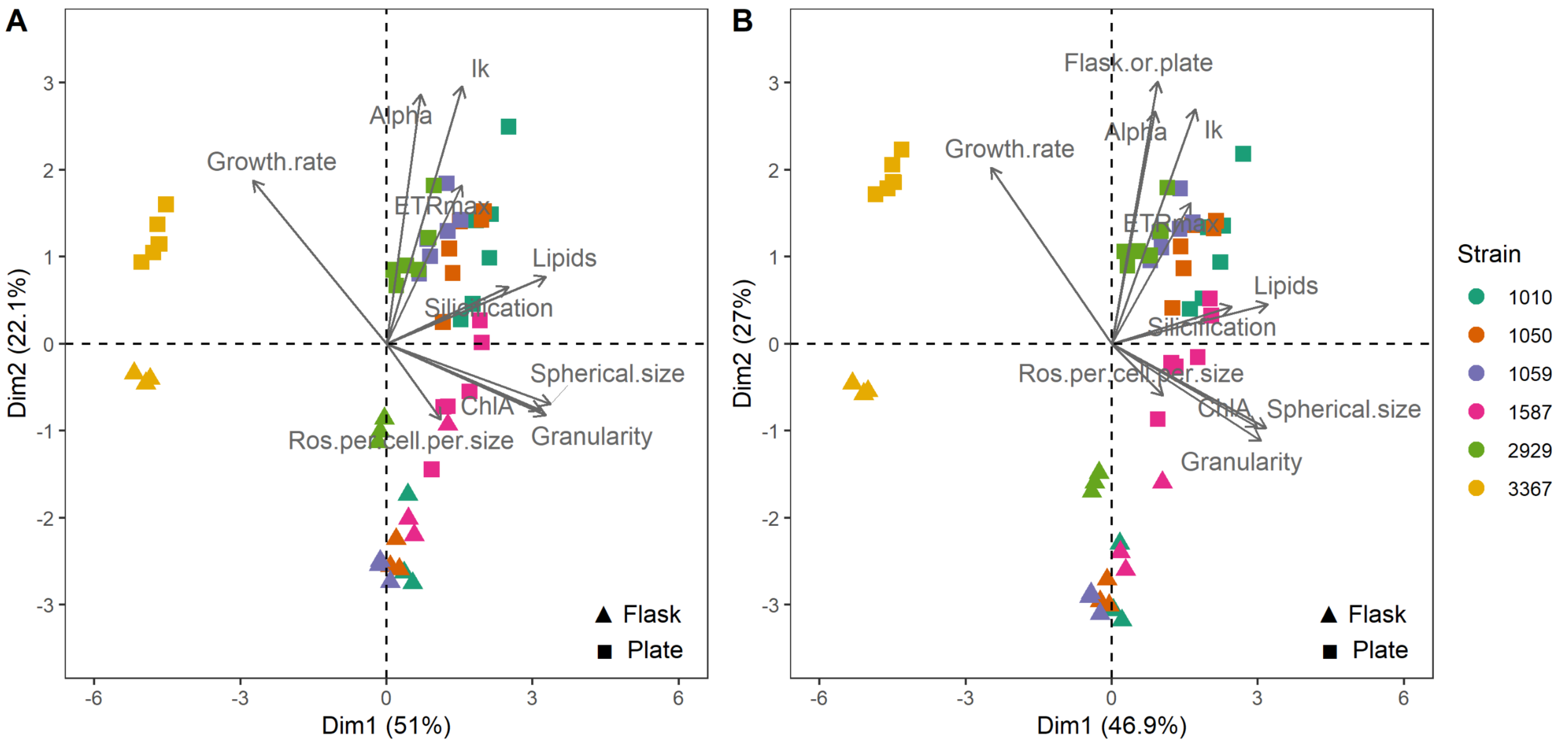

Supplementary Figure S3. PCA analysis of trait data from *Thalassiosira* strains (colours) grown in either 50 mL tissue culture flasks (triangles) or 12-well tissue culture plates (squares). **A** shows the analysis where input variables were the 10 traits used in the QPA. **B** is the same dataset, but with an additional variable of 'Flask or plate' included.

Supplementary Table T3. Raw trait values (mean ± S.E.) for the five centric diatom isolates made from Port Hacking Marine Station grown in natural seawater (NSW) with f/20 nutrients, and artificial seawater (ASW) with f/2 nutrients. T5 did not grow in artificial seawater so is not included for this media.

NSW f/20	alpha	I_k	ETRmax	Growth rate (divisions d^{-1})	Cell Size (µm)	Granularity	Chl-a	Lipids	ROS (*10^{-3})
T1	0.284 ± 0.0049	200 ± 4.6	705 ± 28	1.18 ± 0.026	7.20 ± 0.15	8269 ± 247	19488 ± 501	3874 ± 322	5.1 ± 0.49
T2	0.282 ± 0.0087	212 ± 9.0	755 ± 56	1.21 ± 0.021	7.01 ± 0.11	8269 ± 475	18461 ± 297	3612 ± 149	8.5 ± 0.82
T3	0.261 ± 0.0125	210 ± 9.0	807 ± 46	1.21 ± 0.011	6.99 ± 0.08	8162 ± 81	18367 ± 77	3466 ± 248	9.6 ± 0.35
T4	0.289 ± 0.0049	206 ± 13.3	713 ± 58	1.21 ± 0.011	7.10 ± 0.03	9057 ± 209	17987 ± 116	4885 ± 1054	4.9 ± 0.32
T5	0.265 ± 0.0051	204 ± 2.4	772 ± 18	0.33 ± 0.012	7.88 ± 0.03	5807 ± 326	18541 ± 236	3906 ± 200	30.5 ± 1.83
**ASW f/2**									
T1	0.265 ± 0.0029	198 ± 2.2	747 ± 16	1.19 ± 0.043	8.83 ± 0.083	5886 ± 36	23878 ± 612	2871 ± 1020	1.1 ± 0.11
T2	0.266 ± 0.0045	216 ± 9.4	812 ± 48	1.28 ± 0.017	8.43 ± 0.062	5217 ± 106	20681 ± 302	5062 ± 265	0.6 ± 0.06
T3	0.264 ± 0.0058	208 ± 7.0	788 ± 40	1.36 ± 0.012	7.93 ± 0.068	4655 ± 51	17789 ± 143	3892 ± 145	1.1 ± 0.42
T4	0.273 ± 0.0038	203 ± 7.1	744 ± 36	1.17 ± 0.021	7.51 ± 0.057	4868 ± 46	16870 ± 60	3919 ± 318	0.2 ± 0.01

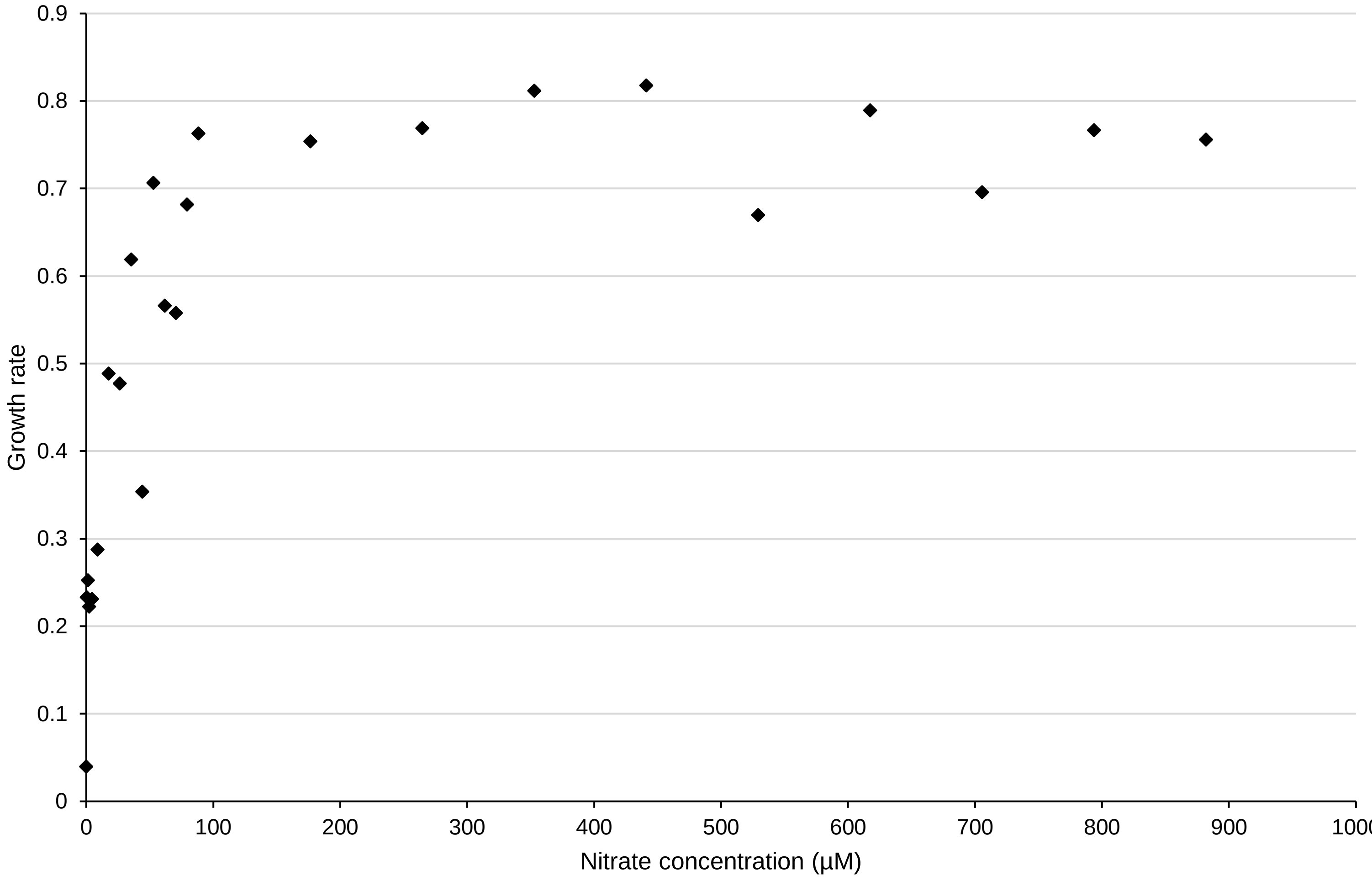

Supplementary Figure S4. Growth rate vs. nitrate concentration for strain CCMP3367 grown in 96-well tissue culture plates. Strains were grown in artificial seawater with f/2 nutrients with variable nitrate concentration with 60 µmol $m^{-2}s^{-1}$ light. F/2 media has 882 µM of nitrate which was the highest concentration used. This was a pilot study thus n = 1 per concentration. Growth rate was not affected even with 8x less nitrate than full media, showing that nitrate is not limited in f/2 media. With <100 µM nitrate growth decreased sharply to a minimum of 0.04 with no nitrate, likely indicating some cell division from stored nitrate. The growth rate at the lowest added nitrate was 0.23 with 0.441 µM nitrate.
